# First but not second postoperative day growth hormone assessments as early predictive tests for long-term acromegaly persistence

**DOI:** 10.1007/s40618-021-01553-0

**Published:** 2021-04-10

**Authors:** V. Cambria, G. Beccuti, N. Prencipe, F. Penner, V. Gasco, F. Gatti, M. Romanisio, M. Caputo, E. Ghigo, F. Zenga, S. Grottoli

**Affiliations:** 1 Division of Endocrinology, Diabetes and Metabolism, Department of Medical Sciences, University of Turin, Corso Dogliotti 14, 10126 Turin, Italy; 2 Division of Neurosurgery, Department of Neurosciences “Rita Levi Montalcini”, University of Turin, Turin, Italy; 3 Division of Endocrinology, Department of Translational Medicine, University of Eastern Piedmont “Amedeo Avogadro”, Novara, Italy

**Keywords:** Growth hormone, Acromegaly, Disease persistence, Early prediction

## Abstract

**Purpose:**

Postoperative assessment of acromegaly activity is typically performed at least 3 months after neurosurgery (NS). Few studies have evaluated the use of early postoperative growth hormone (GH) levels as a test to predict short- and long-term remission of acromegaly. Our objective was to evaluate the diagnostic performance of serum random GH on a postoperative day one (D1-rGH) and two (D2-rGH), particularly in predicting long-term disease persistence.

**Materials and methods:**

Forty-one subjects with acromegaly who were undergoing NS were enrolled (mean age ± SD 47.4 ± 13.1 years at diagnosis; women 54%; macroadenomas 71%). The final assessment of disease activity was performed one year after NS. ROC curves were used to evaluate the diagnostic performance of D1-rGH and D2-rGH.

**Results:**

After a 1-year follow-up, the overall remission rate was 55%. ROC analysis identified an optimal D1-rGH cut-off value of 2.1 ng/mL for diagnosing long-term disease persistence (55.6% SE; 90.9% SP). The cut-off point became 2.5 ng/mL after maximizing specificity for disease persistence (yielding a 100% positive predictive value) and 0.3 ng/mL after maximizing sensitivity for disease remission. The optimal D2-rGH cut-off value was 0.6 ng/mL (81.8% SE; 50% SP); the cut-off point became 2.9 ng/mL after maximizing specificity and 0.1 ng/mL after maximizing sensitivity, with no clinical utility.

**Conclusions:**

D1-rGH could be a highly specific test for the early diagnosis of long-term acromegaly persistence, which is predicted by a value > 2.5 ng/mL with a great degree of certainty. The diagnostic performance of D2-rGH was insufficient. Further research is required to validate these preliminary results prior to modifying the postoperative management of acromegaly.

## Introduction

Neurosurgery (NS) is considered the first choice to cure acromegaly. In the literature, the remission rate is variable (56 − 72%) depending on the clinical center and operator [1]. Moreover, the probability of remission is different between micro- (> 85%) and macroadenomas (40 − 50%) [2]. Technical progress has improved surgical outcomes to achieve a post-NS remission rate of 60 − 85% in the largest cohorts [3, 4].

Re-evaluation of the disease state after NS is typically performed by measuring IGF1 and nadir GH after an oral glucose tolerance test (OGTT) at least 12 weeks post-intervention due to the long half-life of IGF1 [2, 5].

The American Association of Clinical Endocrinologists (AACE) guidelines for acromegaly recommend that “a fasting GH level be measured early postoperatively” to predict long-term remission (recommendation n. 47, “best evidence” level 2, Grade C) [6]. In fact, the short half-life of GH could allow the early prediction of the disease state. Soon after NS, GH levels decrease as a result of the ischemia and fibrosis of residual adenomatous tissue or adenoma removal; the only source of GH production should be normal somatotropic cells, which are still suppressed by the previously high levels of GH and IGF1 [7].

Postoperative cortisol is a recognized predictor of Cushing disease remission, while in acromegaly, the use of postoperative GH has not yet been fully explored.

Some studies have attempted to assess the prognostic role of early random GH determination with different cut-off levels and times, as well as other early postoperative biochemical tests (mean GH, IGF1, nadir GH after OGTT); however, many pitfalls must be considered to avoid incorrect decisions. In fact, in comparison with GH, the decline in IGF1 is delayed due to the longer half-life of IGF-binding proteins. Nadir GH after an OGTT may slightly change over time, from immediately postoperatively to 3 months after NS [8]. A prospective study evaluated the predictive role of random GH and IGF1 on a postoperative day one and nadir GH following an OGTT 2 − 4 days after NS [9]. The authors showed that early determination of IGF1 was not useful for predicting disease state; however, the proposed cut-offs for random GH and nadir GH appeared to demonstrate good positive predictive values for remission but inadequate specificity [9].

Early random GH could be considered a simple and cheap test that is entirely feasible for the postoperative management of acromegaly; a specific and reproducible cut-off point is needed to discriminate between cured patients and those with a high risk of persistent or recurrent disease.

The present study aimed to evaluate the diagnostic performance of GH determination on postoperative days one and two for the early prediction of acromegaly persistence at 1 year after pituitary surgery.

## Materials and methods

We prospectively recruited 41 consecutive subjects with acromegaly who were undergoing NS for pituitary adenoma at the Division of Neurosurgery of the “City of Health and Science of Turin” University Hospital (Turin, Italy) between 2014 and 2018. All surgical procedures were performed by a single neurosurgeon dedicated to pituitary surgery (FZ), using a 3D endoscopic endonasal approach; only one patient needed a combined transcranial and endonasal approach due to a “supergiant” GH-secreting pituitary adenoma [10].

Serum IGF1 was assessed at diagnosis and three months and one year after NS. Serum random GH (rGH) was assessed on day one, day two, and three months after NS. Nadir GH after an OGTT (nGH) was measured three months after NS; adequate inhibition of GH after the OGTT was defined as nGH < 1.0 ng/ml.

Contrast-enhanced magnetic resonance imaging was performed three months after NS.

Acromegaly persistence at the end of the one-year follow-up was defined as the use of medical therapy or the need for radiation therapy due to disease persistence or recurrence after NS, or an IGF1 value above the age-dependent upper limit of normal (ULN) of the Italian population [11], expressed as both absolute value and IGF1 times ULN (IGF1 × ULN); on the contrary, disease remission was defined by an IGF1 value below the age-dependent ULN of the Italian population, without active medical therapy or exposure to radiation therapy.

Serum GH levels (ng/mL) were measured using an immunoradiometric assay (IRMA GH, Beckman Coulter, Czech Republic), and serum IGF1 levels (ng/mL) were measured by a radioimmunoassay technique (SM-C-RIA-CT, DIAsource ImmunoAssays, Belgium) following acid–ethanol extraction to avoid interference of binding proteins.

Data are presented as the mean ± standard deviation (SD), or the median (interquartile range) for skewed variables. Statistical analysis was performed using the Stata program (version 14; Stata Corporation, College Station, TX, USA). The Mann − Whitney *U* test was employed for continuous variables, whose non-normal distribution was attributed to the limited sample size, and the chi-square test was used for categorical variables.

Receiver Operating Characteristics (ROC) analysis was carried out to identify the D1- and D2-rGH cut-off values with the optimal sensitivity/specificity pairing, the highest specificity for acromegaly persistence, and the highest sensitivity for disease remission.

## Results

The demographic and clinical characteristics of the participants are summarized in Table 1. Of the 41 subjects, 54% were women. The mean age was 47.4 ± 13.1 years at diagnosis and 49.0 ± 12.5 years at NS. All patients were affected by a GH-secreting pituitary adenoma (71% macroadenomas) with a median diameter of 12 mm (8 − 21).

**Table 1:** Demographic and clinical features of the study population

Characteristics	All subjects (*n* = 41)
Gender	
Women, *n* (%)	22 (54)
Men, *n* (%)	19 (46)
Age, years	47.4 ± 13.1
Neoadjuvant therapy, *n* (%)	17 (42)
Adenoma diameter, mm	12 (8 − 21)
Microadenoma, *n* (%)	12 (29)
Macroadenoma, *n* (%)	29 (71)
Residual, *n* (%)	14 (34)
Microadenoma, *n* (%)	4 (29)
Macroadenoma, *n* (%)	10 (71)
IGF1, ng/mL; IGF1 (x ULN)	
At diagnosis	848 (546 − 1105); 2.9 (1.9 − 3.7)
Three months after NS	249 (194.5 − 424); 0.9 (0.7 − 1.5)
One year after NS	226 (162 − 364); 0.7 (0.6 − 1.2)
Random GH, ng/mL	
Postoperative day one	1.4 (0.9 − 2.4)
Postoperative day two	0.9 (0.3 − 1.7)
Three months after NS	1.0 (0.4 − 3.8)
Nadir GH after OGTT, ng/mL	
Three months after NS	0.3 (0.1 − 0.8)

Data are presented as the mean ± SD or the median (interquartile range)

At diagnosis, the median IGF1 was 848 ng/mL (546 − 1105) and the median IGF1 (x ULN) was 2.9 (1.9 − 3.7). Neoadjuvant therapy with a somatostatin analog was given to 42% of patients and discontinued at least 3 months prior to NS to avoid pharmacological interference in subsequent biochemical assessments.

Three months after NS, a residual adenoma was found in 34% of patients by contrast-enhanced magnetic resonance imaging. Among these, 71% had originally been affected by macroadenomas, but in comparison with microadenomas, the higher percentage was not statistically significant.

On postoperative day one (D1), the median rGH (D1-rGH) was 1.4 ng/mL (0.9 − 2.4; *n* = 41) and on postoperative day two (D2), the median rGH (D2-rGH) was 0.9 ng/mL (0.3 − 1.7; *n* = 30). After 3 months, the median IGF1 was 249 ng/mL (194.5 − 424; *n* = 40) and the median IGF1 (x ULN) was 0.9 (0.7 − 1.5); 65% of the values were within the reference range. The median rGH was 1.0 ng/mL (0.4 − 3.8; *n* = 38) and the median nGH was 0.3 ng/mL (0.1 − 0.8; *n* = 36); 77.8% of subjects achieved an adequate inhibition of GH after the OGTT. After 1 year, the median IGF1 was 226 ng/mL (162 − 364; *n* = 41), and the median IGF1 (x ULN) was 0.7 (0.6 − 1.2). The overall remission rate was 55%, with no difference between macro- and microadenomas (57% vs. 50%; NS).

Using the ROC curves to predict long-term disease persistence, both sensitivity (SE) and specificity (SP) were optimized for D1-rGH at a cut-off value of 2.1 ng/mL, yielding 55.6% SE, 90.9% SP, a positive likelihood ratio (LH) of 6.11, a negative LH of 0.49, and an area under the curve (AUC) of 0.783 ± 0.075. Three months after NS, among the subjects with a D1-rGH ≤ 2.1 ng/mL, 82.7% showed IGF1 normalization, as well as GH inhibition after the OGTT. Among those with a D1-rGH > 2.1 ng/mL, 16.6% achieved IGF1 normalization and 33.3% reached GH inhibition after the OGTT. The one-year remission rate was 69% among subjects with a D1-rGH ≤ 2.1 ng/mL and 16.6% among those with a D1-rGH > 2.1 ng/mL.

After maximizing specificity for acromegaly persistence, the ROC analysis identified a cut-off value of 2.5 ng/mL (100% SP; 44.4% SE). A D1-rGH > 2.5 ng/mL was observed in eight of the 41 subjects (19.5%), among which 75% were women, 62% had originally been affected by macroadenomas, and 25% did not have a visible residual tumor. Three months after NS, 78.8% of the subjects with a D1-rGH ≤ 2.5 ng/mL showed IGF1 normalization and 84.8% showed GH inhibition after the OGTT. On the contrary, no patients with a D1-rGH > 2.5 ng/mL achieved IGF1 normalization or GH inhibition after the OGTT. One year after NS, 67% of subjects with a D1-rGH ≤ 2.5 ng/mL achieved remission as compared with 0% of those with a D1-rGH > 2.5 ng/mL. The proposed cut-off for the detection of acromegaly persistence after 1 year yielded a 44.4% SE, a 100% SP, a 100% positive predictive value, a 69% negative predictive value, and a 75% accuracy.

**Fig. 1:**
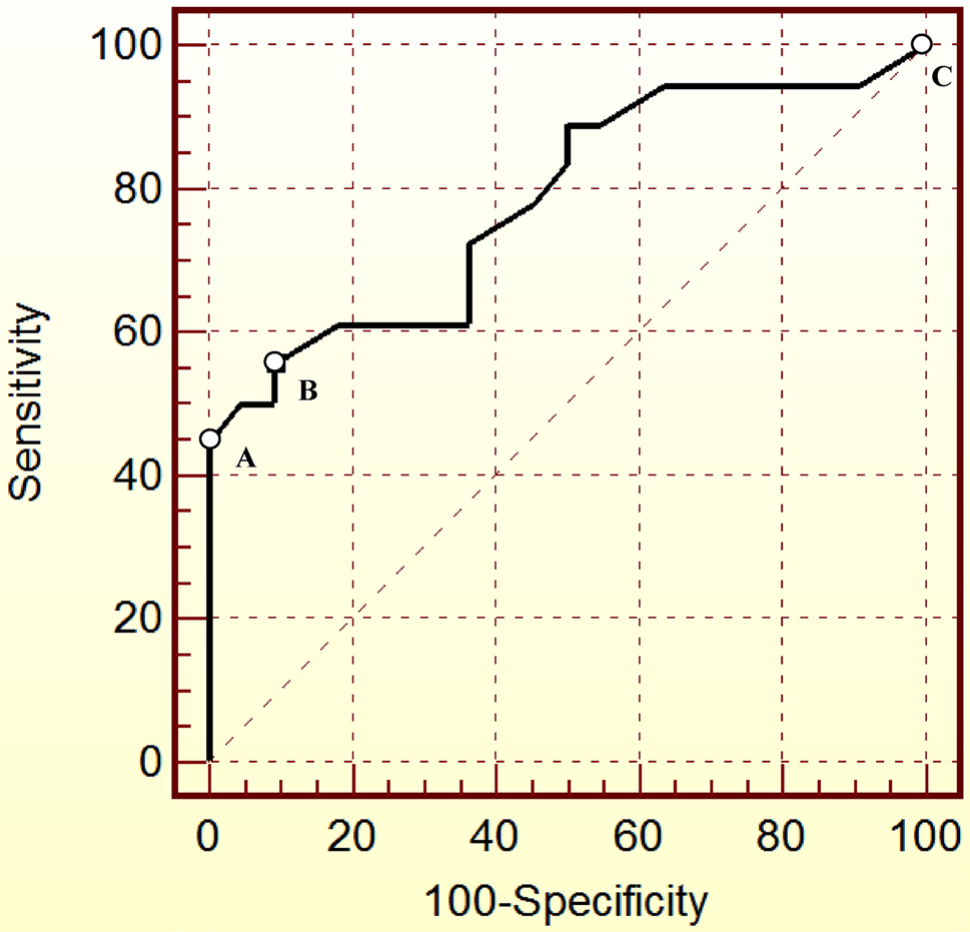
ROC curves showing different cut-off values for random GH on postoperative day one (D1-rGH) for the early prediction of acromegaly persistence 1 year after neurosurgery. **a** Cut-off: 2.5 ng/mL; 44.4% SE; 100% SP; AUC 0.783. **b** Cut-off: 2.1 ng/mL, 55.6% SE; 90.9% SP; AUC 0.783. **c** Cut-off: 0.3 ng/mL; 100% SE; 0% SP; AUC 0.783. *SE *Sensitivity, *SP* Specificity

After maximizing the sensitivity for disease remission, the ROC curves provided a cut-off value of 0.3 ng/mL (100% SE; 0% SP). In our population, only three of the 41 subjects (7%) had a D1-rGH of 0.3 ng/mL, and none had a D1-rGH < 0.3 ng/mL; thus, further statistical analysis was not permitted. The ROC curves displaying different cut-off points for D1-rGH are shown in Fig. 1.

**Fig. 2:**
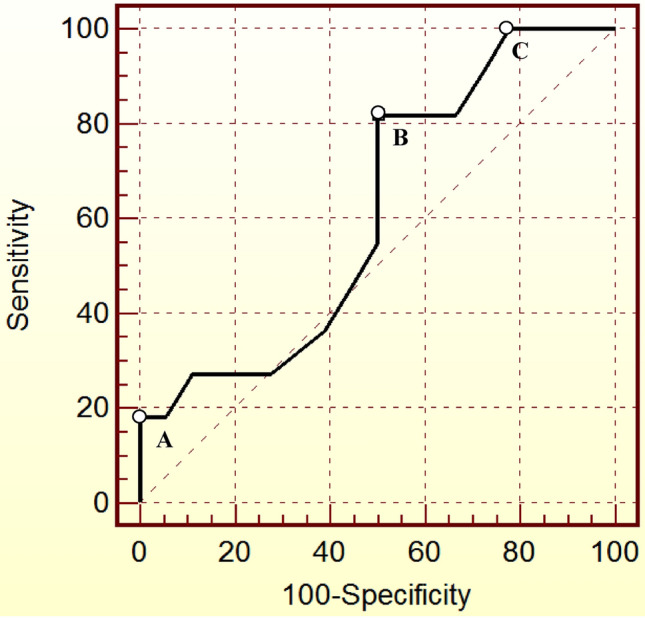
ROC curves showing different cut-off values for random GH on postoperative day two (D2-rGH) for the early prediction of acromegaly persistence 1 year after neurosurgery. **a** Cut-off: 2.9 ng/mL; SE 18.2%; SP 100%; AUC 0.614. **b** Cut-off: 0.6 ng/mL; SE 81.8%; SP 50%; AUC 0.614. **c** Cut-off: 0.1 ng/mL; SE 100%; SP 22.2%; AUC 0.614. *SE* Sensitivity, *SP* Specificity

Similarly, both the SE and SP were optimized for D2-rGH at a cut-off value of 0.6 ng/mL, yielding an SE of 81.8%, an SP of 50%, a positive LH of 1.64, a negative LH of 0.36, and an AUC of 0.614 ± 0.111. After maximizing the specificity for acromegaly persistence, a cut-off point of 2.9 ng/mL was identified (100% SP; 18.2% SE), but a D2-rGH > 2.9 ng/mL was only observed in two of 30 subjects (6.6%). A cut-off point of 0.1 ng/mL yielded a 100% SE and a 22.2% SP, but only four of 30 subjects (13.3%) had a D2-rGH of 0.1 ng/mL, and none had a D2-rGH < 0.1 ng/mL. The ROC curves displaying different cut-off points for D2-rGH are shown in Fig. 2.

## Discussion

This observational study on 41 acromegalic patients suggests that serum random GH on a postoperative day one could be a valid and affordable test for the early prediction of long-term acromegaly persistence. In particular, postoperative day one random GH values > 2.5 ng/mL show a positive predictive value of 100% for disease persistence. On the contrary, serum random GH on postoperative day two does not appear to be a useful predictive factor for either remission or persistence of disease, at least in the present study.

Our population shares the same characteristics as those described in the literature: acromegaly diagnosis in the 5th decade of life, a similar incidence rate between men and women, and macroadenomas in more than 2/3 of cases [12]. Moreover, the remission rate one year after surgery was 55% in our sample, which is similar to that reported in previous studies [12]. However, we did not find a difference in remission rate between micro- and macroadenomas, likely due to the relatively small cohort of patients, as well as the adenoma localization, which has not been considered.

The AACE acromegaly guidelines have suggested the early prediction of disease activity, recommending the use of postoperative fasting GH [6]; however, the recommendation n. 47 of the guidelines only has “best evidence” level 2 (evidence obtained from limited outcome data) and Grade C (action based on weak evidence). Based on a single study [7], the AACE guidelines identified a postoperative day one GH cut-off point of 2 ng/mL for remission; 99% of patients with a postoperative GH < 2 ng/mL had clinical evidence of disease remission five years after NS. On the contrary, early prediction of disease activity and early postoperative GH assessment are not even mentioned in the clinical practice guideline of the Endocrine Society [2].

Different studies have attempted to identify early predictors of disease activity, focusing on long-term remission. Intraoperative GH determination was initially proposed, indicating that a reduction in GH levels during the surgical procedure may improve surgical outcomes [13]; however, intraoperative testing is an elaborate and expensive procedure and therefore is not routinely performed [13–15].

The mean of multiple GH measurements between 1 and 3 weeks after NS appeared to be a promising high-sensitivity test for the prediction of a successful long-term surgical outcome; in fact, a mean GH value < 3 ng/mL predicted disease remission in 100% of cases [16]. These interesting results, however, required multiple clinic admissions, whereas a single early GH evaluation can be easily performed during the patient’s hospitalization.

Some studies have attempted to analyze the use of an early nadir GH after an OGTT. Although OGTT results become more accurate three months after NS [17], many authors have concluded that an early OGTT has a good positive predictive value for acromegaly remission [18, 19]. For example, an OGTT in the first week after NS yielded an 81.7% SE and a 95.1% SP in diagnosing long-term remission [7]. Sarkar et al. evaluated both random GH on postoperative day one and nadir GH after an OGTT on day seven; the optimal cut-off for random GH was 3.66 ng/mL (93.1% SE; 79.2% SP) and for nadir GH was 2.55 ng/mL (93.1% SE; 77.4% SP) [20]. Similar to the mean of multiple measurements, an OGTT on day seven could also lead to time- and cost-consuming hospital readmission. Moreover, OGTT cannot be performed in subjects with diabetes.

Postoperative day one random GH values < 2 ng/ml were highly predictive of long-term remission of acromegaly in a large retrospective study [21]. Other studies have analyzed the diagnostic performance of early random GH with different cut-off points and days of collection. Kim et al. measured random GH on the first three postoperative days after NS and found that day one was the best predictor of acromegaly remission [7]. Dutta et al. assessed random GH six hours post-NS and on days one to five after surgery; GH values ≤ 1.5 ng/mL at six hours showed the highest specificity (83.3%) and ≤ 1.03 ng/mL on day one showed the best sensitivity (90.5%) [22]. The authors also found that the diagnostic performance of other time points was lower, with day four being the worst. Similarly, in our study, random GH on day one appeared to be a better predictor of disease status than that on day two.

Contrarily, Antunes et al. found that GH levels < 1.57 mcg/L on day two yielded a 93% SE and an 86% SP in diagnosing short-term IGF1 normalization. However, this study including 69 patients do not provide data on long-term remission [23].

We note that many diagnostic tests described in the literature are not highly specific for acromegaly remission, since normal post-surgery GH levels could also be due to low disease activity. Therefore, switching our perspective, we used the ROC curves to detect patients with a higher probability of acromegaly persistence.

The optimal sensitivity/specificity cut-off value for the day one random GH yielded a low sensitivity and a high specificity for disease persistence. After maximizing specificity, we identified a cut-off point with a positive predictive value of 100% but with the limitation of a small sample above the cut-off.

The clinical implications of these results are relevant since we were able to identify patients with a very high risk of disease persistence, who most likely would not benefit from intermediate hormonal assessments, i.e., 3 months after pituitary surgery. Based on the limited results of our study, we cannot recommend any disease management alternative to the international guidelines, but we hypothesize that in the future, an early confirmation of disease activity may guide endocrinologists through clinical algorithms including early reintervention or medical therapy without delay.

The main limitation of our study is the relatively small sample size. However, larger studies had a retrospective approach; moreover, when compared with prospective studies, we consecutively recruited in a shorter time and in a single-center, focusing on long-term outcomes too. So, our results need to be confirmed in a larger cohort of patients; in particular, their reproducibility should be evaluated in other neurosurgical centers similar to ours, where a neurosurgeon with expertise in the surgical management of pituitary adenomas is available, as seen in the Italian survey on pituitary surgery [24]. It should be noted that the lack of uniformity of GH and IGF1 assays across different centers could lead to heterogeneous and non-reproducible data. As soon as a large sample size will be prospectively achieved, it would be interesting to employ a machine learning (ML) algorithm to early predict disease persistence, based not only on D1-rGH but also on several variables. To date, few ML-based models have been proposed for predicting off-medication remission with high performance. AUC of the best full model was 0.888 in a training, retrospective cohort of 833 patients (0.871 in the external cohort) [25]; similarly, AUC of the best full model was 0.855 in a training, retrospective cohort of 534 patients (0.817 in the validation cohort) [26].

Interestingly, in comparison with day one, day two random GH had lower diagnostic performance, similar to observations of previous studies. In our case, the main reason was the limited statistical power; however, certain unknown biological mechanisms may be involved, since a time-dependent decrease in diagnostic performance has been observed. A lack of tumor-induced suppression of normal GH secretion and the effects of local factors such as ischemia, fibrosis, cytokines, and inflammation could all play a role in this interesting phenomenon and justify a “sooner the better” approach to postoperative GH scheduling.

In conclusion, we show that random GH on a postoperative day one is a highly specific test for the early prediction of acromegaly persistence one year after pituitary surgery. After maximizing specificity, our test yields a positive predictive value of 100%, and thus predicts long-term disease persistence with a great degree of certainty. On the contrary, the diagnostic performance of random GH on postoperative day two does not demonstrate clinical utility.

We suggest an evaluation of these preliminary data in a broader validation sample, extending the protocol to other pituitary units. It is unclear whether patients with a postoperative day one random GH > 2.5 ng/mL may benefit from an intermediate reassessment of disease status; however, the cut-off value must be validated prior to modifying the postoperative management of acromegaly. Until then, clinicians should follow the recommendations and timings suggested by the international guidelines.
